# Alpha-Difluoromethylornithine, an Irreversible Inhibitor of Polyamine Biosynthesis, as a Therapeutic Strategy against Hyperproliferative and Infectious Diseases

**DOI:** 10.3390/medsci6010012

**Published:** 2018-02-08

**Authors:** Nicole LoGiudice, Linh Le, Irene Abuan, Yvette Leizorek, Sigrid C. Roberts

**Affiliations:** Pacific University School of Pharmacy, Hillsboro, OR 97123, USA; logi8930@pacificu.edu (N.L.); le1143@pacificu.edu (L.L.); abua7932@pacificu.edu (I.A.); leiz1835@pacificu.edu (Y.L.)

**Keywords:** polyamines, ornithine decarboxylase, difluoromethylornithine, eflornithine, DFMO, African sleeping sickness, hirsutism, colorectal cancer, neuroblastoma

## Abstract

The fluorinated ornithine analog α-difluoromethylornithine (DFMO, eflornithine, ornidyl) is an irreversible suicide inhibitor of ornithine decarboxylase (ODC), the first and rate-limiting enzyme of polyamine biosynthesis. The ubiquitous and essential polyamines have many functions, but are primarily important for rapidly proliferating cells. Thus, ODC is potentially a drug target for any disease state where rapid growth is a key process leading to pathology. The compound was originally discovered as an anticancer drug, but its effectiveness was disappointing. However, DFMO was successfully developed to treat African sleeping sickness and is currently one of few clinically used drugs to combat this neglected tropical disease. The other Food and Drug Administration (FDA) approved application for DFMO is as an active ingredient in the hair removal cream Vaniqa. In recent years, renewed interest in DFMO for hyperproliferative diseases has led to increased research and promising preclinical and clinical trials. This review explores the use of DFMO for the treatment of African sleeping sickness and hirsutism, as well as its potential as a chemopreventive and chemotherapeutic agent against colorectal cancer and neuroblastoma.

## 1. Introduction

**Figure 1:**
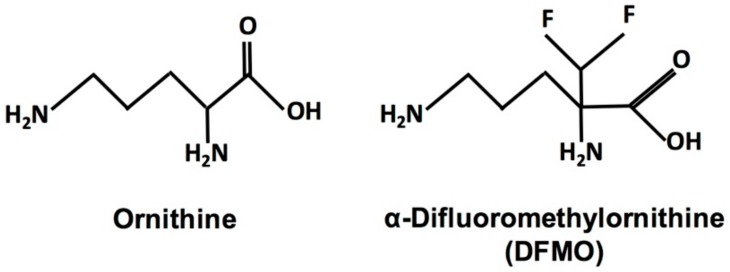
Structures of ornithine and the fluorinated ornithine analog α-difluoromethylornithine (DFMO).

The fluorinated ornithine analog α-difluoromethylornithine (DFMO, eflornithine, ornidyl) (Figure 1) is an irreversible suicide inhibitor of ornithine decarboxylase (ODC), the first and rate-limiting enzyme of polyamine biosynthesis. The compound was developed over 40 years ago as a targeted ODC inhibitor. Polyamines are polycationic aliphatic amines that are ubiquitous and essential for virtually all eukaryotic and prokaryotic cells [1,2,3,4]. The three main polyamines are putrescine, spermidine, and spermine; however, other polyamines exist, and the polyamine biosynthetic pathway and polyamine composition vary between species [1,4]. Polyamines have numerous functions, including, but not limited to, replication, protein biosynthesis, transcription, signal transduction, cell cycle progression, apoptosis, and stress resistance [5,6,7]. However, cellular proliferation is emerging as one of the most prominent functions. While quiescent cells show low ODC activity and putrescine levels, actively dividing cells exhibit increased ODC activity [8,9,10,11]. Enzyme activity is high during late G1 phase before DNA synthesis begins and again in the G2/M transition phase [8,9,10,11]. An essential downstream function of the polyamine pathway is the hypusination and activation of eukaryotic initiation factor eIF5A, vital for protein synthesis and cellular proliferation [12]. Furthermore, multiple studies have found elevated polyamine levels in tumor cells and that inhibition of polyamine pathway enzymes interferes with cellular proliferation [1,11,13,14].

The activity of ODC is highly regulated with multiple mechanisms. ODC is rapidly turned over within the cell by an antizyme-mediated process based on intracellular polyamine concentrations [14]. In addition, regulation of transcription and translation of *ODC* affects the amount of enzyme being expressed [14]. It has been found that ODC activity is increased and polyamines are present at higher concentrations in some solid tumors [11,14]. This has led to ODC inhibition being investigated as a therapeutic strategy for various malignancies, although long-term inhibition of this enzyme is challenging due to the rapid turnover and tight regulation of ODC production.

This review will provide an overview of the mechanism of action and pharmacokinetic properties of DFMO and summarize its clinically approved use against African sleeping sickness and hirsutism. In addition, the promise of DFMO to combat colon cancer and neuroblastoma will be discussed. 

## 2. Pharmacodynamic and Pharmacokinetic Properties of DFMO

DFMO was designed as an enzyme-activated irreversible inhibitor of ODC [15]. The compound is decarboxylated by the enzyme and covalently binds to ODC [16]. The irreversible inhibition of ODC results in depletion of putrescine and spermidine; however, only incomplete depletion of spermine occurs [11]. 

**Figure 2:**
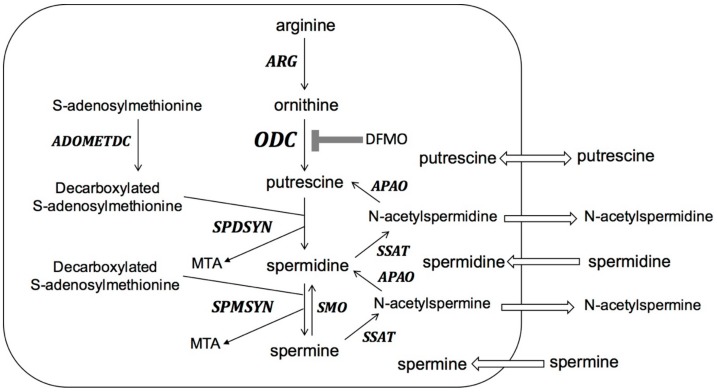
The polyamine pathway in mammalian cells. ARG: arginase; ODC: ornithine decarboxylase; SPDSYN: spermidine synthase; SPMSYN: spermine synthase; ADOMETDC: S-adenosylmethionine decarboxylase; SMO: spermine oxidase; SSAT: spermidine/spermine N1-acetyltransferase; APAO: N-acetyl polyamine oxidase; MTA: methylthioadenosine. Polyamine biosynthesis through ARG, ODC, SPDSYN, SPMSYN and ADOMETDC is shown. Back-conversion from spermine to spermidine occurs through the action of SMO and back-conversion from spermine to spermidine and putrescine is catalyzed by SSAT and APAO. Export of putrescine and acetylated polyamines and import of polyamines from the extracellular milieu are indicated. DFMO inhibition of ODC is shown.

Biosynthesis of polyamines (Figure 2) starts with the conversion of L-arginine to ornithine. The rate-limiting step of polyamine biosynthesis is the conversion of ornithine to putrescine by ODC. Putrescine is further converted to spermidine and spermine by the sequential actions of spermidine synthase (SPDSYN) and spermine synthase (SPMSYN). The aminopropyl group for spermidine and spermine synthesis is donated from decarboxylated S-adenosylmethionine, which is formed from S-adenosylmethionine by the action of S-adenosylmethionine decarboxylase (ADOMETDC). In mammalian cells, a back-conversion pathway exists where spermine oxidase (SMO) converts spermine to spermidine and the combined actions of spermidine/spermine N^1^-acetyltransferase (SSAT) and N-acetyl polyamine oxidase (APAO) convert spermine to spermidine and putrescine. Putrescine and acetylated polyamines can be exported, and polyamines can be imported by the cell. 

Currently, the Food and Drug Administration (FDA) approves DFMO for female facial hirsutism and human African trypanosomiasis or sleeping sickness [10,17,18,19,20,21,22]. Vaniqa (Allergan, Irvine, CA, US) is a 13.9% DFMO cream available for hirsutism with a prescription [10,18,20]. In addition, DFMO has orphan drug status for a variety of cancers including: neuroblastoma, colon, gastric, and pancreatic cancer. DFMO is available as oral, intravenous, and topical formulations. The oral bioavailability of DFMO is approximately 50% for both the solution and tablet formulations [23]. Very little protein binding occurs, and DFMO can cross the blood brain barrier. The serum half-life is 1.5–5 h and 80% of it is excreted in the urine [17]. The rapid elimination from the blood makes high treatment doses necessary, and these poor pharmacokinetic properties render the drug less than ideal [24,25]. For the topical cream Vaniqa, less than 1% of the dose is absorbed systemically and the elimination half is 8 h [26,27].

The DFMO dose in colon cancer prevention trials is typically around 500 mg/m^2^/day DFMO daily in tablet form over several years [28,29,30,31]. Higher oral doses are tested clinically for neuroblastoma treatment in children; 2000 mg/m^2^/day DMFO alone or up to 9000 mg/m^2^/day in combination therapies [32]. For the treatment of African sleeping sickness, DFMO is given intravenously at 400 mg/kg/day for 14 days as monotherapy or 800 mg/kg/day for seven days in combination with Nifurtimox [17,33]. The topical formulation of DFMO in Vaniqa is 13.9%.

At doses between 1–3 g/m^2^/day, DFMO has minimal side effects including anemia, mild gastrointestinal upset, and reversible ototoxicity [10]. Because the drug has been found to reduce fetal body weight and may induce skeletal variations, DFMO is labeled pregnancy category C and should be used with caution [10]. The overall good safety profile has led to DFMO being considered as long-term chemoprevention or adjunct to chemotherapy.

## 3. African Sleeping Sickness

The parasites that give rise to African sleeping sickness are *Trypanosoma brucei rhodesiense* and *Trypanosoma brucei gambiense*. The bites of the tsetse flies transmit infectious trypanosomes to humans and livestock primarily seen in sub-Saharan Africa. The parasite invades the blood, lymph, and ultimately the central nervous system (CNS). The infection may present as acute or chronic depending on the subspecies. *T. b. rhodesiense* give rise to an acute disease state while *T. b. gambiense* are usually responsible for a chronic condition. If left untreated, both forms of the disease may lead to death commonly due to meningoencephalitis. There are no current vaccines available to prevent African sleeping sickness, and only four drugs approved for treatment: suramin, pentamidine, melarsoprol, and DFMO (eflornithine) [17,21,22]. 

Suramin and pentamidine are equally effective in treating early-stage gambiense disease but are less effective with the rhodesiense form [17,22]. Their effectiveness is limited to the early-stage of the disease mainly due to poor cerebrospinal fluid (CSF) penetration. For late-stage manifestations involving the CNS, melarsoprol is the only agent that can treat both subspecies, *T. b. rhodesiense* and *T. b. gambiense* [17,34]. Melarsoprol is a trivalent arsenical compound with weak CSF penetration but effective trypanocidal activity. The drawback to melarsoprol is the significant toxicity and irritation at the injection site. Serious reactions such as arsenic encephalopathy occurs in 10% of patients and are fatal in about half of those cases [17,34]. On top of the high risk for toxicity, a large percentage of non-responders have been observed suggesting the emergence of melarsoprol resistance [17,33,35]. 

**Figure 3:**
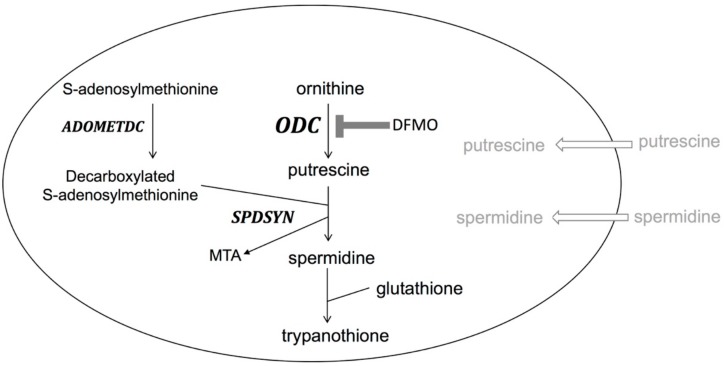
The polyamine pathway in *Trypanosoma brucei. Trypanosoma brucei* parasites lack ARG, SPMSYN, and a back-conversion pathway. Unique to trypanosomatids is the formation of trypanothione, a conjugate of spermidine and glutathione. Polyamine import is minimal as transport capacities are poor and levels of polyamines in blood are low. DFMO inhibition of ODC is shown.

The fourth drug, DFMO, is effective against both early- and late-stage gambiense disease and was approved by the FDA in 1990 [4,17,19,21,22,34]. DFMO has been shown to be the safer alternative to melarsoprol and became the preferred first-line treatment for late-stage gambiense disease [17,19,21,22,34]. The drug effectively crosses the blood–brain barrier and enters the CSF to irreversibly inhibit the parasite’s ODC enzyme [17,19,21,22,34]. Interestingly, the affinity of DFMO to parasite and human ODC is similar and the drug inactivates both enzymes effectively [21]. However, the human enzyme is rapidly turned over (half-life less than an hour), while the parasites ODC has a much longer half-life (over 6 h), thus polyamine biosynthesis in trypanosomes is selectively more affected by the suicide inhibitor than the human host [3,36]. The inhibition of ODC by DFMO leads to the loss of polyamine biosynthesis crucial for parasite replication and survival. Genetic obliteration of ODC by gene deletion or knockdown confirmed that polyamine biosynthesis is essential for parasites [37,38]. Polyamines are especially important in *T. brucei* as, in a reaction unique to trypanosomatids, spermidine is conjugated with glutathione to produce trypanothione, which is essential for maintaining the intracellular redox balance and for defense against oxidative stress [2,3,4]. Furthermore, the polyamine biosynthetic pathway of *T. brucei* is significantly different from that of the mammalian host (Figure 3). Parasites do not contain a functional arginase to synthesize ornithine, the immediate substrate of ODC, do not synthesize or utilize spermine, and have no back-conversion pathway [4]. *T. brucei* parasites reside as extracellular parasites in the bloodstream. Because blood contains only nanomolar concentrations of polyamines and polyamine uptake is poor in trypanosomes, the parasites depend completely on endogenous biosynthesis [4]. This observation, together with the fact that ODC is a stable enzyme in parasites, explains the efficacy of DFMO for the treatment of *T. b. gambiense*. However, DFMO is not effective against *T. b. rhodesiense*; the reason for this is not well understood, but may possibly be due to the rapid disease progression or a higher ODC activity and turnover [3,39].

Although DFMO was originally developed as an anti-cancer agent, its first clinical use was against African trypanosomes. Studies by Cyrus Bacchi and coworkers showed that the compound eliminated parasites in mice and that putrescine biosynthesis was reduced in parasites [40]. A first field trial in Sudan using oral DMFO showed effectiveness in 1985 [41] and subsequent studies using intravenous administration of DFMO were promising [25,42]. In fact, DFMO was so successful in treating patients, even in the comatose later stages of the disease, that the nickname “resurrection drug” was coined [19,43]. However, DFMO has high manufacturing costs and had to be administered at large doses (400 mg/kg/day, 4 times daily for 1–2 weeks) with an average cost per patient of $700 at the time [44]. Consequently, the use of DFMO in Africa waned in the 1990. Aventis (Schiltigheim, France), who had acquired the company originally manufacturing DFMO, sold left-over DFMO to Médecins Sans Frontières and gave the patent rights to the World Health Organization [19]. In an interesting development, DFMO was approved as the active ingredient in Vaniqa, a hair removal face cream, in 2000. The CBS show 60 min aired a segment on the use of DFMO for vanity contrasting this to the unavailability of the drug in Africa to save lives. In the end, the scandal resulted in Aventis producing and providing DFMO for use in Africa [19]. 

The standard dosing regimen of DFMO is intravenous infusions of 100 mg/kg every 6 h for a total of 14 days [17]. This dosing scheme is notably labor intensive, costly, and difficult to administer in developing countries with limited health care resources. In addition, although DFMO is tolerated well, some dose-dependent side effects were noted, such as reversible ototoxicity [18]. Attempts to make the dosing regimen shorter or to administer oral DFMO were not efficacious [17]. To make treatment more manageable, nifurtimox, a synthetic nitrofuran compound with antiprotozoal activity, was added in combination to DFMO therapy. The nifurtimox eflornithine combination therapy (NECT) demonstrated similar efficacy and significantly reduced the amount of DMFO, lessened adverse effects, and shortened treatment duration [17,21,45]. In combination therapy, DFMO is dosed 400 mg/kg/day by intravenous route every 12 h for seven days with concurrent nifurtimox 15 mg/kg/day by oral route every eight hours for 10 days [33]. NECT is listed as one of the essential medicines by the World Health Organization [17,21,45]. Relapse of the disease may occur more than 12 months after treatment; therefore, patient follow-up is recommended [45]. 

DFMO is also effective against a variety of other parasites [4]. For example, in *Leishmania* spp., DFMO has been found to reduce parasite burden in mice and hamsters [46,47,48] and an *ODC* gene deletion mutant was found to be virtually non-infective in mice [49]. Thus, DFMO may have potential against other parasitic diseases as well. 

## 4. Vaniqa

Vaniqa is a DFMO 13.9% cream that is FDA approved since 2000 for unwanted facial hair in women. Unwanted hair growth, or hirsutism, is a significant problem in an estimated 40% of women in the US leading to decreased self-esteem, confidence, and increased social avoidance [20,50]. The hair follicle (HF), an organ composed of 20 different cell types, is highly proliferative and expresses high ODC activity, although the exact function of polyamines for HF is unknown [9]. Early DFMO trials in patients suffering from African sleeping sickness noted reversible hair loss [9,51], which may have spurred the development of DFMO for Vaniqa. Interestingly, the commonly noted side effect of reversible ototoxicity with DFMO is most likely due to impairment of hair cells of the organ of Corti in the inner ear being affected [18,52,53]. Transgenic mouse models have shown marked hair loss when DFMO was administered [54]. Other genetically manipulated mouse models have also confirmed a connection between polyamine metabolism and hair growth, although they show evidence of a more complicated relationship between putrescine and hair growth [9,55,56,57].

Clinical trials with Vaniqa demonstrated significant reduction in unwanted hair in the chin and upper lip area [20,50]. In one study, 58% in the DFMO group were rated as improved, compared to 34% in the control group [50]. Another trial included patient self-assessment, inquiring about the level of bother and discomfort in social gatherings or work due to the skin condition [20]. Again, DFMO resulted in significant benefits compared to the vehicle control [20]. One early study was performed with [^14^C]-labeled DFMO to study percutaneous absorption and pharmacokinetics. Labeled DFMO was not detected in plasma and in only small amounts in urine, demonstrating very limited absorption of topical DFMO [27]. Vaniqa is tolerated well, with low rates of the most common side effects: acne, redness or stinging of the skin [10,50].

The cream must be applied twice daily, with marked improvements seen at week 8 and continued improvements seen at week 24 [50]. This treatment is not a permanent solution for hirsutism; Vaniqa must be continued indefinitely or hair will regrow within two months [50]. DFMO application also increases the effectiveness of laser therapy, which is commonly used for the removal of unwanted hair [50,58].

Various studies have looked at using Vaniqa for pseudofolliculitis barbae (persistent irritations from shaving) and chemoprevention for skin cancer [9,10]. Furthermore, clinical trials found that 10% DFMO ointment reduced the number of actinic keratosis with a concomitant reduction in spermidine levels in the treated skin [59,60]. 

## 5. Colon Cancer

Colorectal cancer (CRC) is the second leading cause of cancer-related deaths in the United States and the third most common cancer in men and in women (www.cdc.org). The slow development of CRC and common genetic alterations involved in disease progression warrant the use of chemopreventive measures to reduce its incidence. Although polyamine production is usually tightly controlled by multiple mechanisms in mammalian cells, dysregulation is often observed in cancer cells [13,28,61,62,63,64]. In general, elevated levels of polyamines are frequently associated with cancers, and higher polyamine concentrations were detected in colon polyps compared to normal mucosa [13,65,66]. Increased activity of ODC and higher levels of polyamines, mainly putrescine and spermidine, have been found in CRC cell lines, rodent models, and human tissue samples [13,28,61,62,63,64]. Thus, the ability of DFMO to suppress polyamine content in CRC is of particular interest, although the molecular mechanism by which polyamines contribute to cancer are not well understood [67].

**Figure 4:**
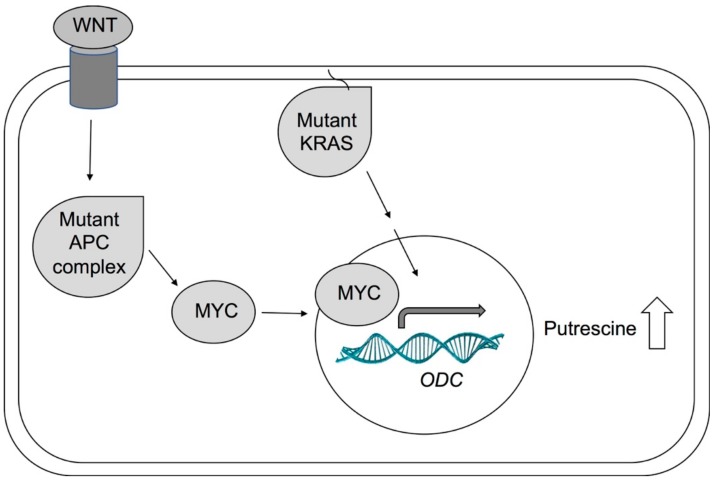
Simplified model of ODC activation in cancer. Activated WNT signaling and APC mutations lead to activation of MYC. The transcription factor MYC travels into the nucleus and induces transcription of several genes, including *ODC*. Mutated *KRAS* can also lead to increased *ODC* expression. In cancers, *APC*, *MYC*, or *KRAS* are often mutated, leading to increased ODC expression and activity and elevated levels of putrescine.

Commonly mutated genes in colon cancer are *APC* tumor-suppressor gene, *KRAS* proto-oncogene, and *MYC* proto-oncogene (Figure 4). *APC* is mutated in familial adenomatous polyposis (FAP), an inherited CRC syndrome, and in over 80% of sporadic colon cancer [28,65]. In normal cells, APC inhibits the proto-oncogene and transcription factor MYC and loss of MYC inhibition results in overexpression of ODC and polyamine synthesis [28,65,68]. Activation of MYC is associated with almost 70% of CRC [13]. *KRAS* is another proto-oncogene and important intracellular signaling molecule. Mutation and activation of *KRAS* lead to increased ODC expression and decreased expression of spermidine/spermine N^1^-acetyltransferase 1 (SSAT), resulting in decreased polyamine catabolism and export [65,68]. ODC activity was found to be upregulated in KRAS mutant cancer cells and DFMO treatment prevented tumor formation in nude mice injected with KRAS activated tumors [69]. The study found that DFMO-mediated prevention of tumorigenesis may occur via altered cell–cell communication [69]. Intriguingly, DFMO treatment prevented occurrence of *KRAS* mutations in a CRC mouse model, which suggests that DFMO is effective in suppressing colon carcinogenesis in the chemopreventive setting [70]. Similarly, animal models have shown that DFMO appears to be especially effective in preventing the transition of noninvasive to invasive cancers [13].

Using metabolomics to examine cellular changes, several metabolites were found to be significantly altered by DFMO treatment in the human colorectal adenocarcinoma cell line HT-29, including a decrease in putrescine, spermidine, methylthioadenosine, and N-acetylputrescine [71]. A separate metabolomic study found that DFMO treatment in ApcMin mice caused a significant reduction in thymidine pools, presumably due to the link of polyamine metabolism to S-adenosylmethionine pathways [72]. The authors conjecture that DFMO mediated ODC inhibition may enhance S-adenosylmethionine decarboxylase activity and thus exhaust levels of S-adenosylmethionine, resulting in impaired methionine cycling and thymidine pools. Indeed, the authors go as far as to imply that the reduction in thymidine pools may be the underlying mechanism of DFMO colon cancer prevention [72].

Depletion of polyamines leads to a reduction in transcription factors and pluripotency factors required for expression of several proteins and enzymes essential to cell cycle progression [67]. DFMO-treated HCT116 colon cancer cells showed lower levels of transcription factor HMGA2 and pluripotency factor LIN28 [67]. Growth inhibition was rescued by exogenous putrescine supplementation, confirming that the growth reducing effects of DFMO are due to polyamine depletion in the colon cancer cells [67]. Given the importance of the growth-associated factors in neoplasia, these findings suggest that polyamines are oncometabolites that influence tumorigenesis by regulating specific growth factors, and thus leading to the increase in tumor numbers and tumor growth rates [67].

Although DFMO is not FDA-approved for the indication of CRC prevention yet, several clinical trials have shown that DFMO alone significantly alters metabolites in colon cancer cells [13,63,64,65,73]. Furthermore, the use of DFMO in combination with other agents for chemopreventive CRC therapy has shown promising results. As inflammation is typically associated with cancer and polyamines have been linked to inflammation-induced carcinogenesis, nonsteroidal anti-inflammatory drugs (NSAID) are potential candidates for combination therapy [28,65]. Cyclooxygenase 1 and 2 (COX-1 and COX-2), the target of NSAIDs, are critical enzymes in inflammatory pathways and both, COX-1 and COX-2, are induced by *APC* mutations [70]. In addition to being anti-inflammatory agents, NSAIDs have been shown to increase polyamine catabolism by inducing SSAT and polyamine export [28,62,65,68]. Thus, the combination of polyamine synthesis inhibitors and anti-inflammatory agents are particularly promising as cancer preventive agents. However, while NSAIDs have been associated with reduced colon cancer rates, they also increase the risk of gastrointestinal and/or cardiovascular side effects, therefore establishing safety profiles is important. Inhibitors of COX-1 and COX-2 (aspirin, sulindac, and piroxicam) and a selective inhibitor of COX-2 (celecoxib) have been advanced for combination therapy with DFMO [28,61,62,65,68]. Aspirin and piroxicam in combination with DFMO have been tested in animal studies and showed promising results [74,75]. A phase II clinical trial with aspirin and DFMO combination therapy to test for adenoma recurrence is underway [76]. The NSAIDs sulindac and celecoxib have been tested in animals and in clinical trials in combination with DFMO with encouraging outcomes [28,29,30,31,61,62,68,70,77,78]. When combined with sulindac, the effect of DFMO on reducing colon tumor numbers and total intestinal polyamine content was enhanced in a azoxymethane-induced colon cancer rat model [70]. Similar results were found in clinical trials [28,30,31,77,78]. A clinical trial with a low dose of 500 mg oral DMFO and 150 mg oral sulindac daily showed impressive efficacy in preventing adenomas in high risk patients with a history of resected adenomas [30,62,78]. The study found a 70% reduction in adenomas, a 92% decrease in advanced adenomas, and a 95% decrease in recurrence of more than one adenoma [30,62,78]. Furthermore, no increased cardiovascular events were observed and a follow up study that specifically investigated the risk of cardiovascular complications confirmed that the combination of DFMO and sulindac did not increase adverse cardiovascular outcomes in patients with normal risk for heart disease [31]. A currently ongoing clinical trial examines the safety and efficacy of the combination of oral 750 mg DFMO and 150 mg sulindac in patients with FAP [28]. A clinical trial testing celecoxib alone and in combination with DFMO found a higher reduction of adenoma count and burden for the combination than for celecoxib alone [29]. The combination treatment reduced adenoma count by 13% compared to 1% with celecoxib alone, and adenoma burden was reduced by 40% with the combination treatment compared to a 27% reduction with the NSAID alone [29]. Both sulindac and celecoxib monotherapy have been used off-label in FAP patients with some efficacy [28]. 

One important aspect to consider is that polyamines and polyamine precursors like arginine can also be taken up by diet. An increased intake in red meat, which is high in arginine, has been shown to decrease overall survival of colon cancer patients [65,77]. Thus, dietary recommendations may be a useful addition to any DFMO-based chemoprevention strategy [65,77].

There is evidence that genetic polymorphism in the *ODC* genomic locus has influence on the effectiveness of DFMO and NSAIDs [61,62,79]. A single nucleotide polymorphism (SNP) in the *ODC* promoter region has been associated with higher efficacy of aspirin for the risk reduction of adenoma recurrence in animals and in clinical trials [79,80]. It is likely that the variation in the promoter region makes *ODC* more susceptible for overexpression in some cancers and thus inhibition of polyamine synthesis by DFMO and/or NSAIDs is particularly effective in cancer prevention in patients exhibiting this polymorphism. The genetic marker may be a diagnostic tool to screen for high risk of cancer development and to identify patients that would receive the highest benefit from chemoprevention [80]. 

Another study examined rosuvastatin as an alternative to NSAIDs for combination therapy with DFMO in mice [81]. Statins, although mostly known for their inhibition of cholesterol synthesis, also have growth modulatory and anti-inflammatory effects and have been promoted as potential chemopreventive agents [81]. In addition, rosuvastatin appears to inhibit arginase activity and polyamine biosynthesis, thus DFMO and rosuvastatin may have an additive effect in decreasing polyamine levels [81]. Azoxymethane-induced colon cancer rat models were dosed individually and in combination with 500 ppm DFMO and 50 ppm rosuvastatin in food [81]. Rosuvastatin plus DFMO suppressed colon adenocarcinoma multiplicity by 76% compared to rosuvastatin monotherapy, 29%, and DFMO monotherapy, 46%, suggesting additive effects [81]. As expected, the treated colon tumors exhibited decreased polyamine content. Furthermore, they exhibited increased intratumoral natural killer (NK) cells expressing perforin plus interferon (IFN)-γ compared with untreated colon tumors [81]. This boost of innate immune cells in colon tumors may provide enhanced cytotoxic effects that can trigger lysis of the tumor cells, thus preventing progression of colon adenocarcinomas [81].

In these chemopreventive clinical trials, oral DFMO is typically given at 500 mg/m^2^/day, with few side effects [13,29,30,31,64,78]. A particular concern with DFMO treatment is reversible ototoxicity [82,83]; however, only some dose-dependent reversible ototoxicity was observed while most trials reported no ototoxicity [13,29,30,31,64,78]. The low levels of both DFMO and NSAIDs required for cancer prevention compared to treatment correlate to the observed low levels of toxicity.

In summary, evidence from in vitro studies, animal models, and clinical trials demonstrate that DFMO mediated ODC inhibition causes metabolic changes, in particular polyamine depletion, which, in turn, decreases cellular proliferation and increases carcinogenesis suppression. Combination treatment of DFMO with NSAIDs and possibly other agents like rosuvastatin are even more effective and, although not yet FDA approved, are promising approaches for the chemoprevention of CRC. 

## 6. Neuroblastoma

The term neuroblastoma is used to describe a range of tumors affecting immature nerve cells and accounts for 97% of these tumors. This cancer is fast spreading and can be found anywhere on the sympathetic nervous system, although 40% is found on the adrenal gland. The majority of patients affected by this cancer are children under four years of age. If diagnosed before the age of 1, patients have favorable outcomes. Patients older than one with advanced stage disease have a more negative, often lethal outcome stemming from progressive disease despite intensive multimodality therapy [32,84,85]. About 13% of pediatric cancer mortality can be attributed to neuroblastoma [86]. 

Genomic analyses in neuroblastoma tumors have shown that *MYC* mutations are present in about 40% of high risk tumors that show poor prognosis [32,87]. Evidence from a transgenic tyrosine hydroxylase (TH) promoter-driven mouse model (TH-MYCN) confirms that *MYC* is a driver gene as these mice develop peripheral neural tumors with complete penetrance in homozygous mice [88,89]. As observed in other cancers, and described above for colon cancer, the *ODC* gene is a target of MYC and neuroblastoma tumors often show high ODC activity [32,89]. Importantly, the ODC locus maps within 5 Mb of *MYC*, and both genes are often co-amplified [32]. Studies have linked inferior outcomes with higher expression of several polyamine biosynthetic enzymes and lower expression of catabolic enzymes in patients and animal models [32,84,90]. Indeed, *ODC* and other polyamine enzyme-encoding genes might be useful as diagnostic markers, independent of *MYC* [32]. High ODC expression has also been found in high risk tumors even without *MYC* mutations, confirming that ODC is a bona fide target [90]. 

Because MYC has proven notoriously ‘undruggable’ [91] and high tumor ODC activity has been linked to lower survival in cancer patients [32,90], inhibition of ODC by DFMO is a promising therapeutic strategy. Indeed, DFMO administration prevents tumor formation in the hemizygous TH-MYCN mouse and delays tumor onset in the homozygous mouse [90]. In vitro studies with neuroblastoma cell lines found that DFMO inhibited proliferation and reduced colony formation in a dose-dependent manner [32]. In human MYCN-amplified neuroblastoma cell lines, inhibition of ODC led to increased expression of cyclin-dependent kinase inhibitor p27Kip1, retinoblastoma protein (Rb) hypophosphorylation, and cell cycle arrest [92]. In TH-MYCN mice DFMO administration resulted in an increased expression of cyclin-dependent kinase inhibitor 1, p21^Cip1^, a protein normally repressed in neuroblastoma, also resulting in cell cycle arrest and prevention of tumor cell migration and invasion of tumor cells [85,89]. New studies in the TH-MYCN mouse model suggest that polyamine depletion also has an effect on the tumor microenvironment and may modulate the immune response to prevent tumor proliferation [32].

The combination of DFMO with several other agents has shown even more promise. Early studies found that the reduction of polyamine biosynthesis by combined inhibition of ODC and ADOMETDC by DFMO and methylglyoxal-bis-guanylhydrazone (MGBG), respectively, was more potent than single agent treatment in reducing tumor formation in a rat prostate cancer model [93]. Compound SAM486 was developed based on the MGBG pharmacophore and has proven to be more efficacious than DFMO alone in extending survival in the TH-MYCN neuroblastoma mouse model [94]. DFMO treatment resulted in reduction of putrescine levels but not spermidine and spermine [90], while inhibition with DFMO and SAM486 showed reduced levels of all three polyamines [94]. Thus, the effectiveness of the drug combination can be rationalized by a more effective overall depletion of polyamines. Similar to observations in CRC chemopreventive trials, the addition of celecoxib also increased the effectiveness of DFMO in TH-MYCN mice [94]. This could be due to celecoxib’s anti-inflammatory ability and/or its effect on polyamine catabolism. Furthermore, combination therapy with DFMO enhances the effect of the traditional chemotherapy agents, such as vincristine, cyclophosphamide, cisplatin, and topotecan in TH-MYCN mice [90,94].

Polyamine uptake is increased in DFMO treated cells and polyamine supplementation rescues the inhibitory effect of DFMO suggesting that scavenging of surrounding polyamines could negate the consequences of DFMO treatment [95,96,97]. It is likely that the early disappointing clinical trials in the 1970s and 1980s could at least in part be attributed to monotherapy with DFMO causing increased uptake of polyamines [1,98,99,100,101,102,103]. New approaches that combined the inhibition of polyamine synthesis and uptake, coined polyamine-blocking therapy (PBT), are effective in melanoma cell lines and in transgenic mice. Furthermore, this strategy resulted in an inhibition of the local tumor immunosuppressive response [99,100,104]. The dual impact of PBT on inhibition of polyamine dependent proliferation and as an immunostimulatory strategy may be particularly promising in anticancer therapy [99]. Indeed, recent studies confirmed that the combined strategy is also successful in neuroblastoma, as well as prostate, pancreatic, and breast cancer cell lines [102,105,106,107]. 

Numerous clinical trials have been initiated with children suffering from neuroblastoma. A completed phase I clinical trial investigated DFMO with and without the topoisomerase inhibitor etoposide with the main goal to assess drug safety [85]. Children with refractory or recurrent neuroblastoma were given between 500–1500 mg/m^2^ DFMO orally twice a day. DFMO was given for three weeks as monotherapy followed by DFMO plus etoposide. No dose-limiting or drug related adverse side effects were observed. The study also evaluated the effect of the SNP rs2302616 in the *ODC* locus and found that this variant was associated with enhanced ODC susceptibility and responsiveness to DFMO therapy [85]. Two phase II clinical trials are currently underway to investigate the safety and efficacy of DFMO monotherapy to prevent recurrence of neuroblastoma that is in remission (NCT01586260 and NCT02395666). A similar Neuroblastoma Maintenance Therapy Trial is also ongoing (NCT 02679144). A phase I study is testing different doses of DFMO in combination with celecoxib, cyclophosphamide, and topotecan (NCT02030964) and another ongoing trial investigates the combination of DFMO with bortezimib for relapsed or refractory neuroblastoma (NCT 02139397). In yet another trial, DFMO will be used for two years as maintenance care after treatment with targeted therapy (NCT 02559778).

Although treatment with DFMO against cancer had been overall disappointing in the past, preclinical studies in the TH-MYCN neuroblastoma mouse model and clinical trials in children with this devastating disease are promising. Neuroblastoma may be especially responsive to DFMO treatment because *MYC* mutations and increased ODC activity are so prevalent in this type of cancer. Indeed, further genotyping a patient’s tumor for *MYC* mutations, or ideally ODC overexpression, may improve clinical success of DFMO even more. In addition, not just high ODC activity but also genetic polymorphism within the *ODC* locus appears to be predictive of DFMO treatment success for both CRC prevention and neuroblastoma treatment. As with all new cancer strategies in the era of precision medicine, analyzing the molecular and genetic characteristics of a patient’s tumor will be key to determine if any given drug strategy might be successful.

It should be noted that DFMO has also been advanced as a chemotherapeutic strategy for cancers other than colorectal cancer and neuroblastoma. For example, pancreatic cancer is an intriguing target, as the pancreas has the highest levels of spermidine of any human tissue [108,109]. Studies showed that DFMO is effective in preventing pancreatic cancer in genetically engineered KRAS mice [110] and furthermore that the combination therapy of ODC and polyamine transport inhibition in pancreatic cancer cell lines and pancreatic cancer mouse models inhibited cancer cell survival and prolonged the survival of tumor bearing mice [106,108]. Other areas of prolific research include but are not limited to skin, endometrial, and breast cancer [111,112,113,114,115,116]. Clinical trials with DFMO are ongoing for prostate cancer, skin cancer and precancerous conditions, gastric cancer, bladder cancer, the rare brain cancer anaplastic astrocytoma, cervical cancer, and esophageal cancer (www.clinicaltrials.gov). In summary, this resurgence of DFMO as an investigative cancer preventive or cancer treatment agent is remarkable.

## 7. Conclusions

DFMO targets a strange assortment of diseases, from parasitic infections to hirsutism to cancer. However, the common underlying molecular mechanism of all these afflictions is hyperproliferation. African trypanosomes are rapidly proliferating protozoan parasites, hirsutism is caused by aberrant and increased hair follicle growth, and the basic definition of cancer is uncontrolled proliferation. Polyamines have emerged as crucial metabolites for rapidly proliferating cells and DFMO inhibits ODC, the first and rate-limiting enzyme in this pathway. Although these ubiquitous and essential cations play many roles, their vital importance for cellular proliferation is now taking center stage in research. 

The history of DFMO is intriguing as it was originally developed as an anticancer drug and is now experiencing a resurgence for exactly that purpose. Paradoxically though, the drug is currently not FDA approved for cancer prevention or treatment, but rather for an infectious disease and unwanted hair growth. Here, DFMO serves as an impressive example for drug development for neglected tropical diseases. With no economic incentives, DFMO production for Africa came to a halt and only the conscience of affluent countries reinvigorated its production. DFMO is also an intriguing drug as it has poor pharmacokinetic parameters, and modern drug development would probably not advance this chemical today. It is surprising that, despite much research, no other ODC inhibitors have been developed for clinical use, which elevates DFMO as an important and successful compound. Indeed, recently several clinical trials—with more underway—show that DFMO has a good safety profile and promising efficacy as chemopreventive or chemotherapeutic agent against colorectal cancer, neuroblastoma, and other hyperproliferative diseases.

In summary, DFMO has proven tremendously beneficial for numerous disease states and the recent surge in basic research and preclinical and clinical trials promises more future benefits. 

## References

[1] Gerner E.W., Meyskens F.L. (2004). Polyamines and cancer: Old molecules, new understanding. Nat. Rev. Cancer.

[2] Heby O., Persson L., Rentala M. (2007). Targeting the polyamine biosynthetic enzymes: A promising approach to therapy of African sleeping sickness, Chagas’ disease, and leishmaniasis. Amino Acids.

[3] Heby O., Roberts S.C., Ullman B. (2003). Polyamine biosynthetic enzymes as drug targets in parasitic protozoa. Biochem. Soc. Trans..

[4] Roberts S., Ullman B. (2017). Parasite Polyamines as Pharmaceutical Targets. Curr. Pharm. Des..

[5] Miller-Fleming L., Olin-Sandoval V., Campbell K., Ralser M. (2015). Remaining Mysteries of Molecular Biology: The Role of Polyamines in the Cell. J. Mol. Biol..

[6] Igarashi K., Kashiwagi K. (2010). Modulation of cellular function by polyamines. Int. J. Biochem. Cell Biol..

[7] Bachrach U., Wang Y.C., Tabib A. (2001). Polyamines: New cues in cellular signal transduction. News Physiol. Sci..

[8] Rai P.R., Somani R.R., Kandpile P.S. (2017). Ornithine Decarboxylase Inhibition: A strategy to combat various diseases. Mini Rev. Med. Chem..

[9] Ramot Y., Pietila M., Giuliani G., Rinaldi F., Alhonen L., Paus R. (2010). Polyamines and hair: A couple in search of perfection. Exp. Dermatol..

[10] Smith K.J., Skelton H. (2006). α-Difluoromethylornithine, a polyamine inhibitor: Its potential role in controlling hair growth and in cancer treatment and chemo-prevention. Int. J. Dermatol..

[11] Wallace H.M., Fraser A.V. (2004). Inhibitors of polyamine metabolism: Review article. Amino Acids.

[12] Park M.H., Nishimura K., Zanelli C.F., Valentini S.R. (2010). Functional significance of eIF5A and its hypusine modification in eukaryotes. Amino Acids.

[13] Meyskens F.L., Gerner E.W. (1999). Development of difluoromethylornithine (DFMO) as a chemoprevention agent. Clin. Cancer Res..

[14] Pegg A.E. (2006). Regulation of ornithine decarboxylase. J. Biol. Chem..

[15] Metcalf B.W., Bey P., Danzin C., Jung M.J., Casara P., Vevert J.P. (1978). Catalytic Irreversible Inhibition of Mammalian Ornithine Decarboxylase (E.C.4.1.1.17) by Substrate and Product Analogs. J. Am. Chem. Soc..

[16] Pegg A.E., McGovern K.A., Wiest L. (1987). Decarboxylation of α-difluoromethylornithine by ornithine decarboxylase. Biochem. J..

[17] Babokhov P., Sanyaolu A.O., Oyibo W.A., Fagbenro-Beyioku A.F., Iriemenam N.C. (2013). A current analysis of chemotherapy strategies for the treatment of human African trypanosomiasis. Pathog. Glob. Health.

[18] Coyne P.E. (2001). The eflornithine story. J. Am. Acad. Dermatol..

[19] Ebikeme C. (2014). The death and life of the resurrection drug. PLoS Negl. Trop. Dis..

[20] Jackson J., Caro J.J., Caro G., Garfield F., Huber F., Zhou W., Lin C.S., Shander D., Schrode K., Eflornithine H.S.G. (2007). The effect of eflornithine 13.9% cream on the bother and discomfort due to hirsutism. Int. J. Dermatol..

[21] Jacobs R.T., Nare B., Phillips M.A. (2011). State of the art in African trypanosome drug discovery. Curr. Top. Med. Chem..

[22] Steverding D. (2010). The development of drugs for treatment of sleeping sickness: A historical review. Parasit Vectors.

[23] Carbone P.P., Douglas J.A., Thomas J., Tutsch K., Pomplun M., Hamielec M., Pauk D. (2000). Bioavailability study of oral liquid and tablet forms of α-difluoromethylornithine. Clin. Cancer Res..

[24] Legros D., Ollivier G., Gastellu-Etchegorry M., Paquet C., Burri C., Jannin J., Buscher P. (2002). Treatment of human African trypanosomiasis—Present situation and needs for research and development. Lancet Infect. Dis..

[25] Milord F., Pepin J., Loko L., Ethier L., Mpia B. (1992). Efficacy and toxicity of eflornithine for treatment of *Trypanosoma brucei gambiense* sleeping sickness. Lancet.

[26] Jobanputra K.S., Rajpal A.V., Nagpur N.G. (2007). Eflornithine. Indian J. Dermatol. Venereol. Leprol..

[27] Malhotra B., Noveck R., Behr D., Palmisano M. (2001). Percutaneous absorption and pharmacokinetics of eflornithine HCl 13.9% cream in women with unwanted facial hair. J. Clin. Pharmacol..

[28] Burke C.A., Dekker E., Samadder N.J., Stoffel E., Cohen A. (2016). Efficacy and safety of eflornithine (CPP-1X)/sulindac combination therapy versus each as monotherapy in patients with familial adenomatous polyposis (FAP): Design and rationale of a randomized, double-blind, Phase III trial. BMC Gastroenterol..

[29] Lynch P.M., Burke C.A., Phillips R., Morris J.S., Slack R., Wang X., Liu J., Patterson S., Sinicrope F.A., Rodriguez-Bigas M.A. (2016). An international randomised trial of celecoxib versus celecoxib plus difluoromethylornithine in patients with familial adenomatous polyposis. Gut.

[30] Meyskens F.L., McLaren C.E., Pelot D., Fujikawa-Brooks S., Carpenter P.M., Hawk E., Kelloff G., Lawson M.J., Kidao J., McCracken J. (2008). Difluoromethylornithine plus sulindac for the prevention of sporadic colorectal adenomas: A randomized placebo-controlled, double-blind trial. Cancer Prev. Res..

[31] Zell J.A., Pelot D., Chen W.P., McLaren C.E., Gerner E.W., Meyskens F.L. (2009). Risk of cardiovascular events in a randomized placebo-controlled, double-blind trial of difluoromethylornithine plus sulindac for the prevention of sporadic colorectal adenomas. Cancer Prev. Res..

[32] Bassiri H., Benavides A., Haber M., Gilmour S.K., Norris M.D., Hogarty M.D. (2015). Translational development of difluoromethylornithine (DFMO) for the treatment of neuroblastoma. Transl. Pediatr..

[33] Priotto G., Kasparian S., Mutombo W., Ngouama D., Ghorashian S., Arnold U., Ghabri S., Baudin E., Buard V., Kazadi-Kyanza S. (2009). Nifurtimox-eflornithine combination therapy for second-stage African *Trypanosoma brucei gambiense* trypanosomiasis: A multicentre, randomised, phase III, non-inferiority trial. Lancet.

[34] Priotto G., Pinoges L., Fursa I.B., Burke B., Nicolay N., Grillet G., Hewison C., Balasegaram M. (2008). Safety and effectiveness of first line eflornithine for *Trypanosoma brucei gambiense* sleeping sickness in Sudan: Cohort study. BMJ.

[35] Eperon G., Balasegaram M., Potet J., Mowbray C., Valverde O., Chappuis F. (2014). Treatment options for second-stage gambiense human African trypanosomiasis. Expert Rev. Antiinfect. Ther..

[36] Wang C.C. (1991). A novel suicide inhibitor strategy for antiparasitic drug development. J. Cell. Biochem..

[37] Li F., Hua S.B., Wang C.C., Gottesdiener K.M. (1998). Trypanosoma brucei brucei: Characterization of an ODC null bloodstream form mutant and the action of alpha-difluoromethylornithine. Exp. Parasitol..

[38] Xiao Y., McCloskey D.E., Phillips M.A. (2009). RNA interference-mediated silencing of ornithine decarboxylase and spermidine synthase genes in *Trypanosoma brucei* provides insight into regulation of polyamine biosynthesis. Eukaryot. Cell..

[39] Iten M., Mett H., Evans A., Enyaru J.C., Brun R., Kaminsky R. (1997). Alterations in ornithine decarboxylase characteristics account for tolerance of *Trypanosoma brucei rhodesiense* to d,l-α-difluoromethylornithine. Antimicrob. Agents Chemother..

[40] Bacchi C.J., Nathan H.C., Hutner S.H., McCann P.P., Sjoerdsma A. (1980). Polyamine metabolism: A potential therapeutic target in trypanosomes. Science.

[41] Van Nieuwenhove S., Schechter P.J., Declercq J., Bone G., Burke J., Sjoerdsma A. (1985). Treatment of gambiense sleeping sickness in the Sudan with oral DFMO (DL-α-difluoromethylornithine), an inhibitor of ornithine decarboxylase; first field trial. Trans. R. Soc. Trop. Med. Hyg..

[42] Doua F., Boa F.Y., Schechter P.J., Miezan T.W., Diai D., Sanon S.R., De Raadt P., Haegele K.D., Sjoerdsma A., Konian K. (1987). Treatment of human late stage gambiense trypanosomiasis with α-difluoromethylornithine (eflornithine): Efficacy and tolerance in 14 cases in Cote d’Ivoire. Am. J. Trop. Med. Hyg..

[43] Kuzoe F.A. (1991). Perspectives in research on and control of African trypanosomiasis. Ann. Trop. Med. Parasitol..

[44] Bacchi C.J. (2006). Progress in Anti-Polyamine Drug Development/Chemotherapy vs. Protozoan-Caused Diseases: The DFMO Story. http://wizard.musc.edu/dfmostory.pdf.

[45] Yun O., Priotto G., Tong J., Flevaud L., Chappuis F. (2010). NECT is next: Implementing the new drug combination therapy for *Trypanosoma brucei gambiense* sleeping sickness. PLoS Negl. Trop. Dis..

[46] Gradoni L., Iorio M.A., Gramiccia M., Orsini S. (1989). In Vivo effect of eflornithine (DFMO) and some related compounds on *Leishmania infantum* preliminary communication. Farmaco.

[47] Mukhopadhyay R., Madhubala R. (1993). Effect of a bis(benzyl)polyamine analogue, and DL-α-difluoromethylornithine on parasite suppression and cellular polyamine levels in golden hamster during *Leishmania donovani* infection. Pharmacol. Res..

[48] Olenyik T., Gilroy C., Ullman B. (2011). Oral putrescine restores virulence of ornithine decarboxylase-deficient *Leishmania donovani* in mice. Mol. Biochem. Parasitol..

[49] Boitz J.M., Yates P.A., Kline C., Gaur U., Wilson M.E., Ullman B., Roberts S.C. (2009). *Leishmania donovani* ornithine decarboxylase is indispensable for parasite survival in the mammalian host. Infect. Immun..

[50] Wolf J.E., Shander D., Huber F., Jackson J., Lin C.S., Mathes B.M., Schrode K., Eflornithine H.S.G. (2007). Randomized, double-blind clinical evaluation of the efficacy and safety of topical eflornithine HCl 13.9% cream in the treatment of women with facial hair. Int. J. Dermatol..

[51] Pepin J., Milord F., Guern C., Schechter P.J. (1987). Difluoromethylornithine for arseno-resistant *Trypanosoma brucei gambiense* sleeping sickness. Lancet.

[52] Jansen C., Mattox D.E., Miller K.D., Brownell W.E. (1989). An animal model of hearing loss from α-difluoromethylornithine. Arch. Otolaryngol. Head Neck Surg..

[53] Salzer S.J., Mattox D.E., Brownell W.E. (1990). Cochlear damage and increased threshold in α-difluoromethylornithine (DFMO) treated guinea pigs. Hear. Res..

[54] Wheeler D.L., Ness K.J., Oberley T.D., Verma A.K. (2003). Inhibition of the development of metastatic squamous cell carcinoma in protein kinase C ε transgenic mice by α-difluoromethylornithine accompanied by marked hair follicle degeneration and hair loss. Cancer Res..

[55] Janne J., Alhonen L., Pietila M., Keinanen T.A. (2004). Genetic approaches to the cellular functions of polyamines in mammals. Eur. J. Biochem..

[56] Pietila M., Parkkinen J.J., Alhonen L., Janne J. (2001). Relation of skin polyamines to the hairless phenotype in transgenic mice overexpressing spermidine/spermine N^1^-acetyltransferase. J. Investig. Dermatol..

[57] Soler A.P., Gilliard G., Megosh L.C., O’Brien T.G. (1996). Modulation of murine hair follicle function by alterations in ornithine decarboxylase activity. J. Investig. Dermatol..

[58] Hamzavi I., Tan E., Shapiro J., Lui H. (2007). A randomized bilateral vehicle-controlled study of eflornithine cream combined with laser treatment versus laser treatment alone for facial hirsutism in women. J. Am. Acad. Dermatol..

[59] Alberts D.S., Dorr R.T., Einspahr J.G., Aickin M., Saboda K., Xu M.J., Peng Y.M., Goldman R., Foote J.A., Warneke J.A. (2000). Chemoprevention of human actinic keratoses by topical 2-(difluoromethyl)-DL-ornithine. Cancer Epidemiol. Biomark. Prev..

[60] Bartels P., Yozwiak M., Einspahr J., Saboda K., Liu Y., Brooks C., Bartels H., Alberts D.S. (2009). Chemopreventive efficacy of topical difluoromethylornithine and/or triamcinolone in the treatment of actinic keratoses analyzed by karyometry. Anal. Quant. Cytol. Histol..

[61] Babbar N., Gerner E.W. (2011). Targeting polyamines and inflammation for cancer prevention. Recent Results Cancer Res..

[62] Gerner E.W., Meyskens F.L. (2009). Combination chemoprevention for colon cancer targeting polyamine synthesis and inflammation. Clin. Cancer Res..

[63] Love R.R., Jacoby R., Newton M.A., Tutsch K.D., Simon K., Pomplun M., Verma A.K. (1998). A randomized, placebo-controlled trial of low-dose α-difluoromethylornithine in individuals at risk for colorectal cancer. Cancer Epidemiol. Biomark. Prev..

[64] Meyskens F.L., Gerner E.W., Emerson S., Pelot D., Durbin T., Doyle K., Lagerberg W. (1998). Effect of α-difluoromethylornithine on rectal mucosal levels of polyamines in a randomized, double-blinded trial for colon cancer prevention. J. Natl. Cancer Inst..

[65] Rial N.S., Meyskens F.L., Gerner E.W. (2009). Polyamines as mediators of APC-dependent intestinal carcinogenesis and cancer chemoprevention. Essays Biochem..

[66] Thompson P.A., Wertheim B.C., Zell J.A., Chen W.P., McLaren C.E., LaFleur B.J., Meyskens F.L., Gerner E.W. (2010). Levels of rectal mucosal polyamines and prostaglandin E2 predict ability of DFMO and sulindac to prevent colorectal adenoma. Gastroenterology.

[67] Paz E.A., LaFleur B., Gerner E.W. (2014). Polyamines are oncometabolites that regulate the LIN28/let-7 pathway in colorectal cancer cells. Mol. Carcinog..

[68] Gerner E.W., Meyskens F.L., Goldschmid S., Lance P., Pelot D. (2007). Rationale for, and design of, a clinical trial targeting polyamine metabolism for colon cancer chemoprevention. Amino Acids.

[69] Ignatenko N.A., Zhang H., Watts G.S., Skovan B.A., Stringer D.E., Gerner E.W. (2004). The chemopreventive agent α-difluoromethylornithine blocks Ki-ras-dependent tumor formation and specific gene expression in Caco-2 cells. Mol. Carcinog..

[70] LeGendre-McGhee S., Rice P.S., Wall R.A., Sprute K.J., Bommireddy R., Luttman A.M., Nagle R.B., Abril E.R., Farrell K., Hsu C.H. (2015). Time-serial Assessment of Drug Combination Interventions in a Mouse Model of Colorectal Carcinogenesis Using Optical Coherence Tomography. Cancer Growth Metastasis.

[71] Ibanez C., Simo C., Valdes A., Campone L., Piccinelli A.L., Garcia-Canas V., Cifuentes A. (2015). Metabolomics of adherent mammalian cells by capillary electrophoresis-mass spectrometry: HT-29 cells as case study. J. Pharm. Biomed. Anal..

[72] Witherspoon M., Chen Q., Kopelovich L., Gross S.S., Lipkin S.M. (2013). Unbiased metabolite profiling indicates that a diminished thymidine pool is the underlying mechanism of colon cancer chemoprevention by α-difluoromethylornithine. Cancer Discov..

[73] Meyskens F.L., Emerson S.S., Pelot D., Meshkinpour H., Shassetz L.R., Einspahr J., Alberts D.S., Gerner E.W. (1994). Dose de-escalation chemoprevention trial of α-difluoromethylornithine in patients with colon polyps. J. Natl. Cancer Inst..

[74] Jacoby R.F., Cole C.E., Tutsch K., Newton M.A., Kelloff G., Hawk E.T., Lubet R.A. (2000). Chemopreventive efficacy of combined piroxicam and difluoromethylornithine treatment of APC mutant Min mouse adenomas, and selective toxicity against APC mutant embryos. Cancer Res..

[75] Li H., Schut H.A., Conran P., Kramer P.M., Lubet R.A., Steele V.E., Hawk E.E., Kelloff G.J., Pereira M.A. (1999). Prevention by aspirin and its combination with α-difluoromethylornithine of azoxymethane-induced tumors, aberrant crypt foci and prostaglandin E2 levels in rat colon. Carcinogenesis.

[76] Laukaitis C.M., Erdman S.H., Gerner E.W. (2012). Chemoprevention in patients with genetic risk of colorectal cancers. Colorectal Cancer.

[77] Raj K.P., Zell J.A., Rock C.L., McLaren C.E., Zoumas-Morse C., Gerner E.W., Meyskens F.L. (2013). Role of dietary polyamines in a phase III clinical trial of difluoromethylornithine (DFMO) and sulindac for prevention of sporadic colorectal adenomas. Br. J. Cancer.

[78] Sporn M.B., Hong W.K. (2008). Concomitant DFMO and sulindac chemoprevention of colorectal adenomas: A major clinical advance. Nat. Clin. Pract. Oncol..

[79] Martinez M.E., O’Brien T.G., Fultz K.E., Babbar N., Yerushalmi H., Qu N., Guo Y., Boorman D., Einspahr J., Alberts D.S. (2003). Pronounced reduction in adenoma recurrence associated with aspirin use and a polymorphism in the ornithine decarboxylase gene. Proc. Natl. Acad. Sci. USA.

[80] Hubner R.A., Muir K.R., Liu J.F., Logan R.F., Grainge M.J., Houlston R.S. (2008). Members of the, U.C. Ornithine decarboxylase G316A genotype is prognostic for colorectal adenoma recurrence and predicts efficacy of aspirin chemoprevention. Clin. Cancer Res..

[81] Janakiram N.B., Mohammed A., Bryant T., Zhang Y., Brewer M., Duff A., Biddick L., Singh A., Lightfoot S., Steele V.E. (2016). Potentiating NK cell activity by combination of Rosuvastatin and Difluoromethylornithine for effective chemopreventive efficacy against Colon Cancer. Sci. Rep..

[82] Lao C.D., Backoff P., Shotland L.I., McCarty D., Eaton T., Ondrey F.G., Viner J.L., Spechler S.J., Hawk E.T., Brenner D.E. (2004). Irreversible ototoxicity associated with difluoromethylornithine. Cancer Epidemiol. Biomark. Prev..

[83] Pasic T.R., Heisey D., Love R.R. (1997). α-difluoromethylornithine ototoxicity. Chemoprevention clinical trial results. Arch. Otolaryngol. Head Neck Surg..

[84] Gamble L.D., Hogarty M.D., Liu X., Ziegler D.S., Marshall G., Norris M.D., Haber M. (2012). Polyamine pathway inhibition as a novel therapeutic approach to treating neuroblastoma. Front. Oncol..

[85] Saulnier Sholler G.L., Gerner E.W., Bergendahl G., MacArthur R.B., VanderWerff A., Ashikaga T., Bond J.P., Ferguson W., Roberts W., Wada R.K. (2015). A Phase I Trial of DFMO Targeting Polyamine Addiction in Patients with Relapsed/Refractory Neuroblastoma. PLoS ONE.

[86] Louis C.U., Shohet J.M. (2015). Neuroblastoma: Molecular pathogenesis and therapy. Annu. Rev. Med..

[87] Evageliou N.F., Hogarty M.D. (2009). Disrupting polyamine homeostasis as a therapeutic strategy for neuroblastoma. Clin. Cancer Res..

[88] Rasmuson A., Segerstrom L., Nethander M., Finnman J., Elfman L.H., Javanmardi N., Nilsson S., Johnsen J.I., Martinsson T., Kogner P. (2012). Tumor development, growth characteristics and spectrum of genetic aberrations in the TH-MYCN mouse model of neuroblastoma. PLoS ONE.

[89] Rounbehler R.J., Li W., Hall M.A., Yang C., Fallahi M., Cleveland J.L. (2009). Targeting ornithine decarboxylase impairs development of MYCN-amplified neuroblastoma. Cancer Res..

[90] Hogarty M.D., Norris M.D., Davis K., Liu X., Evageliou N.F., Hayes C.S., Pawel B., Guo R., Zhao H., Sekyere E. (2008). ODC1 is a critical determinant of MYCN oncogenesis and a therapeutic target in neuroblastoma. Cancer Res..

[91] Dang C.V., Reddy E.P., Shokat K.M., Soucek L. (2017). Drugging the ‘undruggable’ cancer targets. Nat. Rev. Cancer.

[92] Wallick C.J., Gamper I., Thorne M., Feith D.J., Takasaki K.Y., Wilson S.M., Seki J.A., Pegg A.E., Byus C.V., Bachmann A.S. (2005). Key role for p27Kip1, retinoblastoma protein Rb, and MYCN in polyamine inhibitor-induced G1 cell cycle arrest in MYCN-amplified human neuroblastoma cells. Oncogene.

[93] Herr H.W., Kleinert E.L., Relyea N.M., Whitmore W.F. (1984). Potentiation of methylglyoxal-bis-guanylhydrazone by α-difluoromethylornithine in rat prostate cancer. Cancer.

[94] Evageliou N.F., Haber M., Vu A., Laetsch T.W., Murray J., Gamble L.D., Cheng N.C., Liu K., Reese M., Corrigan K.A. (2016). Polyamine Antagonist Therapies Inhibit Neuroblastoma Initiation and Progression. Clin. Cancer Res..

[95] Alhonen-Hongisto L., Seppanen P., Janne J. (1980). Intracellular putrescine and spermidine deprivation induces increased uptake of the natural polyamines and methylglyoxal bis(guanylhydrazone). Biochem. J..

[96] Chen Y., Weeks R.S., Burns M.R., Boorman D.W., Klein-Szanto A., O’Brien T.G. (2006). Combination therapy with 2-difluoromethylornithine and a polyamine transport inhibitor against murine squamous cell carcinoma. Int. J. Cancer.

[97] Sunkara P.S., Prakash N.J., Rosenberger A.L. (1982). An essential role for polyamines in tumor metastases. FEBS Lett..

[98] Burns M.R., Graminski G.F., Weeks R.S., Chen Y., O’Brien T.G. (2009). Lipophilic lysine-spermine conjugates are potent polyamine transport inhibitors for use in combination with a polyamine biosynthesis inhibitor. J. Med. Chem..

[99] Hayes C.S., Burns M.R., Gilmour S.K. (2014). Polyamine blockade promotes antitumor immunity. Oncoimmunology.

[100] Hayes C.S., Shicora A.C., Keough M.P., Snook A.E., Burns M.R., Gilmour S.K. (2014). Polyamine-blocking therapy reverses immunosuppression in the tumor microenvironment. Cancer Immunol. Res..

[101] Nowotarski S.L., Woster P.M., Casero R.A. (2013). Polyamines and cancer: Implications for chemotherapy and chemoprevention. Expert Rev. Mol. Med..

[102] Samal K., Zhao P., Kendzicky A., Yco L.P., McClung H., Gerner E., Burns M., Bachmann A.S., Sholler G. (2013). AMXT-1501, a novel polyamine transport inhibitor, synergizes with DFMO in inhibiting neuroblastoma cell proliferation by targeting both ornithine decarboxylase and polyamine transport. Int. J. Cancer.

[103] Casero R.A., Marton L.J. (2007). Targeting polyamine metabolism and function in cancer and other hyperproliferative diseases. Nat. Rev. Drug Discov..

[104] Alexander E.T., Minton A., Peters M.C., Phanstiel O.T., Gilmour S.K. (2017). A novel polyamine blockade therapy activates an anti-tumor immune response. Oncotarget.

[105] Devens B.H., Weeks R.S., Burns M.R., Carlson C.L., Brawer M.K. (2000). Polyamine depletion therapy in prostate cancer. Prostate Cancer Prostatic Dis..

[106] Gitto S.B., Pandey V., Oyer J.L., Copik A.J., Hogan F.C., Phanstiel O., Altomare D.A. (2018). Difluoromethylornithine Combined with a Polyamine Transport Inhibitor Is Effective against Gemcitabine Resistant Pancreatic Cancer. Mol. Pharm..

[107] Muth A., Madan M., Archer J.J., Ocampo N., Rodriguez L., Phanstiel O. (2014). Polyamine transport inhibitors: Design, synthesis, and combination therapies with difluoromethylornithine. J. Med. Chem..

[108] Massaro C., Thomas J., Phanstiel Iv O. (2017). Investigation of Polyamine Metabolism and Homeostasis in Pancreatic Cancers. Med. Sci..

[109] Phanstiel O. (2017). An overview of polyamine metabolism in pancreatic ductal adenocarcinoma. Int. J. Cancer.

[110] Mohammed A., Janakiram N.B., Madka V., Ritchie R.L., Brewer M., Biddick L., Patlolla J.M., Sadeghi M., Lightfoot S., Steele V.E. (2014). Eflornithine (DFMO) prevents progression of pancreatic cancer by modulating ornithine decarboxylase signaling. Cancer Prev. Res..

[111] Bailey H.H., Kim K., Verma A.K., Sielaff K., Larson P.O., Snow S., Lenaghan T., Viner J.L., Douglas J., Dreckschmidt N.E. (2010). A randomized, double-blind, placebo-controlled phase 3 skin cancer prevention study of α-difluoromethylornithine in subjects with previous history of skin cancer. Cancer Prev. Res..

[112] Jeter J.M., Alberts D.S. (2012). Difluoromethylornithine: The proof is in the polyamines. Cancer Prev. Res..

[113] Kreul S.M., Havighurst T., Kim K., Mendonca E.A., Wood G.S., Snow S., Borich A., Verma A., Bailey H.H. (2012). A phase III skin cancer chemoprevention study of DFMO: Long-term follow-up of skin cancer events and toxicity. Cancer Prev. Res..

[114] Kim H.I., Schultz C.R., Buras A.L., Friedman E., Fedorko A., Seamon L., Chandramouli G.V.R., Maxwell G.L., Bachmann A.S., Risinger J.I. (2017). Ornithine decarboxylase as a therapeutic target for endometrial cancer. PLoS ONE.

[115] Arisan E.D., Obakan P., Coker A., Palavan-Unsal N. (2012). Inhibition of ornithine decarboxylase alters the roscovitine-induced mitochondrial-mediated apoptosis in MCF-7 breast cancer cells. Mol. Med. Rep..

[116] Zhu Q., Jin L., Casero R.A., Davidson N.E., Huang Y. (2012). Role of ornithine decarboxylase in regulation of estrogen receptor alpha expression and growth in human breast cancer cells. Breast Cancer Res. Treat..

